# Complications and oncologic outcome in bladder cancer patients receiving radical cystectomy after intravesical instillation treatment

**DOI:** 10.1371/journal.pone.0337644

**Published:** 2025-12-05

**Authors:** Lisa J. Frey, Katarzyna E. Banasiewicz, Christian Ruckes, Isabel Wagner, Maximilian Haack, René Mager, Eva Juengel, Axel Haferkamp, Gregor Duwe, Maximilian P. Brandt

**Affiliations:** 1 Department of Urology and Pediatric Urology, University Medical Center, Mainz, Germany; 2 Interdisciplinary Center for Clinical Trials (IZKS), University Medical Center, Mainz, Germany; Ifo IRCCS Ragina Elena National Cancer Institute, ITALY

## Abstract

**Objective:**

To evaluate the impact of previous intravesical treatment with Bacillus Calmette-Guérin (BCG) or mitomycin on postoperative complications and oncologic outcome in non-muscle invasive bladder cancer (NMIBC) patients receiving radical cystectomy (RC).

**Patients and methods:**

Between 2016 and 2021, 366 patients who received RC for bladder cancer (BC) were screened. Patients with intravesical treatment with either BCG or mitomycin for NMIBC were compared to patients with BC without prior intravesical treatment. Patients were matched manually for ASA-score, BMI cluster, age, and sex. TNM stages were used as covariates in the analysis model. All complications within 30 days post-surgery and the 90-day mortality rates were analyzed. Patient characteristics were compared between groups using Chi^2^-tests, and associations were evaluated by logistic regression. Firth logistic regression was applied for comorbidities. Kaplan–Meier curves were generated for overall survival (OS) and progression-free survival (PFS), and Cox regression analysis was performed to further assess survival outcomes.

**Results:**

We identified 51 matched patients receiving BCG or mitomycin for NMIBC. Nine women and 42 men were in the interventional group with a mean age of 70 and a BMI of 27.6. In both groups, ASA-score 2, 3, and 4 were present in 18, 29, and four patients. According to the Clavien–Dindo (CD) classification grade ≥ 3b, neither the incidence nor severity of complications differed significantly between groups. Final histopathology and survival outcomes (OS, PFS) were comparable. No associations were found between complications and urinary diversion or surgical approach. As expected, advanced T- and N-stages correlated with worse survival, while coronary artery disease (CAD) was significantly associated with postoperative complications.

**Conclusions:**

To our knowledge, this is the first matched pair analysis that evaluates the impact of intravesical treatment on postoperative complications for patients with NMIBC receiving RC. Intravesical treatment with BCG or mitomycin did not deteriorate postoperative complications in NMIBC patients after RC, irrespective of the final histopathology stage. Oncologic outcomes were similar in both groups.

## Introduction

Radical cystectomy (RC) is a standard therapy option for patients with non-muscle invasive bladder cancer (NMIBC) with high-risk or very high-risk pathological features, as well as the gold standard for muscle-invasive bladder cancer (MIBC). However, in patients with intermediate, high, or very high-risk NMIBC, intravesical treatment with Bacillus Calmette Guérin (BCG) or mitomycin represents an alternative option to achieve disease control and reduce the risk of recurrence and progression, therefore avoiding RC. BCG and mitomycin are valid treatment options with clinically proven efficacy from large randomized controlled trials [1,2]. International guidelines recommend intravesical treatment with BCG for at least one year in intermediate or more than one year in high-risk NMIBC patients. Complications associated with intravesical treatment can typically occur locally or systemically. Among these complications are persistent urinary tract inflammation, BCG-induced cystitis or systemic BCG-induced infection, and recurrent macrohematuria [3]. In case of tumor progression, recurrence, or intolerable side effects, RC is the therapy of choice for these patients.

Complications after RC are common in clinical routine and the benefit of early elective RC in NMIBC has to be weighed against the risk of overtreatment. Despite a low 30-day mortality rate of around 1% to 3% [4], complications such as infections, bleeding, ileus formation, or thrombosis occur in up to 40% and are associated with increased morbidity [5]. Considering oncologic outcomes, data from recent publications suggest that cancer-specific and overall survival in very high-risk NMIBC patients are similar in patients treated with BCG compared to early RC alone [6]. On the contrary and importantly, delay in RC for patients with pT1 G3 disease with progression to a stage ≥pT2 is accompanied by adverse oncologic outcomes [7]. In addition to standard-of-care intravesical treatment with BCG or mitomycin, multiple novel treatment options have been recently investigated. Examples are intravesical continuous chemotherapy application with the TAR-system or intravesical gene therapy with a replication-deficient recombinant adenovirus that releases interferon alfa-2b in cancer [8,9]. These treatment options offer new possibilities for patients with NMIBC, thereby preventing or at least delaying RC.

Little evidence exists on the impact of previous local intravesical treatment with BCG or mitomycin on postoperative outcomes of patients with NMIBC with consecutive RC. This study aims to analyze the impact of intravesical treatment with BCG or mitomycin compared to no intravesical therapy on postoperative complications and assess the oncologic outcome between the two groups.

## Materials and methods

### Data and patient selection

A single-center, consecutive database of all patients who received RC for bladder cancer (BC) between 2016 and 2021 was established and maintained. The database was then screened for patients who received intravesical treatment either with BCG or mitomycin for NMIBC. The data were assessed between 01.05.2021 and 01.09.2023 for research purposes. Patients with histologically proven BC, either NMIBC with failure after intravesical treatment or patients with MIBC, were included. Only patients with more than one cycle of intravesical treatment were included in the interventional group. In the non-interventional group, patients with only one instillation of mitomycin (e.g., single post-TUR-B treatment), no intravesical treatment, and primary MIBC were allowed. The current study was approved by the local ethics committee of the Rhineland-Palatinate Medical Association (#2021–15768) and was conducted in accordance with the Declaration of Helsinki and the International Ethical Guidelines for Biomedical Research Involving Human Subjects. Based on the ethics committee’s approval, the requirement for informed consent was waived due to the retrospective nature of the study. All data were pseudonymized, and no third parties had access.

### Assessment of complications and clinical factors

Complications were recorded from digital patient data files and classified according to Clavien-Dindo (CD) [10]. All complications, up to a maximum of six, occurring within the first 30 postoperative days after RC were documented separately. Among all the complications, the most severe complication was analyzed for each patient. In addition, 90-day postoperative mortality was assessed.

### Statistical and matched pair analysis

**Fig 1:**
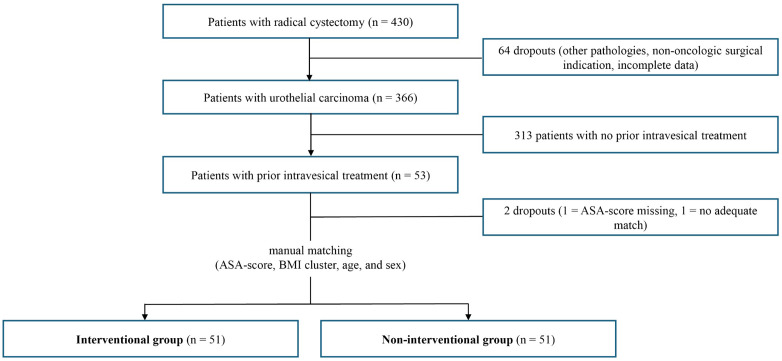
Consort diagram of the matching process.

Matching was performed manually in a 1:1 manner. Primary matching criteria were ASA-score, BMI cluster, age, and sex. All selected criteria are preoperative parameters that can be evaluated independently of the RC procedure and serve as potential indicators relevant to perioperative risk assessment. The BMI clusters were defined as 1 = < 18.5, 2 = 18.5–24.9, 3 = 25.0–29.9, 4 = 30.0–34.9, 5 = 35.0–39.9, and 6 = ≥ 40. Matching criteria were encoded using a structured four-character alphanumeric system applied in a fixed sequence to define each matched pair. The first character determined the interventional or non-interventional group. The second character represented the ASA-score. BMI values were categorized into clusters, encoded as the third character, while the exact BMI was additionally represented by the fourth character of the matching code. This coding system facilitated efficient assignments and helped identify the best counterparts. No deviations were allowed regarding the ASA-score and BMI cluster. A maximum age difference of three years was permitted. If multiple matching partners were available, the smallest BMI difference was prioritized, with same-sex pairs preferred. When more than two potential matches met all criteria equally, random assignment was applied (Table 1). Using this approach, 51 patients in the interventional group (Group A) were matched to 51 patients in the non-interventional group (Group B) (Fig 1). The distribution of matching variables for the full cohort of 366 patients with urothelial carcinoma is provided in the supplementary material (S1 Table).

**Table 1:** Four-character code for the manual matching.

First character	Second character	Third character	Fourth character
Intervention	ASA-score	BMI cluster	BMI
Yes = A No = B	I = A	< 18,5 = A	< 19 = 1
	II = B	[18.5; 24.9] = B	< 20 = 2
	III = C	[25.0; 29.9] = C	< 21 = 3
	IV = D	[30.0; 34.9] = D	...
	V = E	[35.0; 39.9] = E	< 40 = 22
	VI = F	≥ 40.0 = F	≥ 41 = 23

BMI, body mass index; ASA, American Society of Anesthesiologists

Consort diagram illustrating patient selection.

The TNM classification was implemented as a covariate to assess the potential influence of advanced tumor stages on postoperative complications.

The statistical analysis was performed using IBM SPSS Statistics Version 29 (Armonk, NY, USA: IBM Corp.). Continuous variables are presented as mean ± standard deviation (SD) following the data distribution. Categorical and binary baseline parameters were reported as absolute numbers and percentages.

To compare both groups regarding complications and TNM stages, a Chi^2^-test was performed. Associations between complications and both urinary diversion type and surgical approach were evaluated using logistic regression. For the analysis of comorbidities, a multivariable Firth logistic regression was conducted. All statistical tests were two-tailed, and a p-value < 0.05 was considered statistically significant.

Oncologic outcomes, including overall survival (OS) and progression-free survival (PFS), were analyzed using the Kaplan–Meier method and compared with the log-rank test. Kaplan–Meier curves were generated using GraphPad Prism Version 8 (GraphPad Software, Boston, MA, USA). In addition, a Cox proportional hazards regression analysis for OS, including T- and N-stage as covariates, was performed to further assess survival outcomes.

## Results

### Patient characteristics

We identified 51 patients in the interventional and non-interventional group, respectively. In both groups, the mean age was 70 years (SD in both groups: 9.3). The mean BMI was 27.6 (SD: 4.3) in the interventional group and 27.4 (SD: 3.8) in the non-interventional group. Overall, the ASA-score was two in 36 patients, three in 58 patients, and four in eight patients. There were 42 men and nine women in the interventional group and 41 men and ten women in the non-interventional group. In the interventional group, 30 patients (58.8%) received BCG (with a mean number of 11.2 instillations) and 21 (41.2%) patients received mitomycin (with a mean number of 17.1 instillations). An ileal conduit was performed in 78 patients (interventional group: 40; non-interventional-group: 38), sigma conduit in one patient in the interventional group, ureterocutaneostomy in five patients (interventional group: three; non-interventional-group: two), Mainz pouch I in four patients (interventional group: three; non-interventional-group: one), and neobladder in 13 patients (interventional group: three; non-interventional-group: ten). Most patients were treated using an open surgical approach (44 in the interventional group and 48 in the non-interventional group). A robotic-assisted approach was used in only seven cases in the interventional group and three in the non-interventional group. The patient characteristics are listed in Table 2.

**Table 2:** Patient characteristics of both groups.

	Interventional group (n^a^ = 51)	Non-interventional group (n^a^ = 51)
Mean age, years (SD)	70 (9.3)	70 (9.3)
Male, n^a^ (%) Women, n^a^ (%)	42 (82.4) 9 (17.6)	41 (80.4) 10 (19.6)
Mean BMI (SD)	27.6 (4.3)	27.4 (3.8)
BMI Cluster, n^a^ (%)		
2	13 (25.5)	13 (25.5)
3	26 (51)	26 (51)
4	10 (19.6)	10 (19.6)
5	2 (3.9)	2 (3.9)
ASA-score, n^a^ (%)		
2	18 (35.3)	18 (35.3)
3	29 (56.9)	29 (56.9)
4	4 (7.8)	4 (7.8)
Instillation treatment, n^a^ (%)		
BCG	30 (58.8)	–
Mitomycin	21 (41.2)	–
Urinary diversion, n^a^ (%)		
Ileal conduit	40 (78.4)	38 (74.5)
Sigma conduit	1 (2.0)	–
Ureterocutaneostomy	3 (5.9)	2 (3.9)
Neobladder	3 (5.9)	10 (19.6)
Mainz Pouch I	3 (5.9)	1 (2.0)
Other	1 (2.0)	–
Surgical approach n^a^ (%)		
Robotic-assisted RC	7 (13.7)	3 (5.9)
Open RC	44 (86.3)	48 (94.1)

^a^Numbers reflect the number of patients (percentages); SD, standard deviation; BMI, body mass index; ASA, American Society of Anesthesiologists; RC, radical cystectomy.

### Complications

In the first 30 postoperative days, the most common complications in the interventional group were gastrointestinal (23.5%), infectious (21.6%), and cardiopulmonary (15.7%). Twenty-four patients (47.1%) needed a blood transfusion within the first 30 postoperative days. In the non-interventional group, the most common complications were cardiopulmonary (29.4%), gastrointestinal (23.5%), and infections (19.6%). Twenty-one patients (41.2%) received a blood transfusion. The most common complications for both groups are listed in Table 3. Overall, there was no statistically significant difference in the postoperative occurrence of complications between the two groups (p = 0.12). In addition, clinically relevant complications defined as CD ≥ 3b did not reveal significant differences between the two groups (p = 0.8). Regarding the comorbidities (Table 4), only coronary artery disease (CAD) was identified as a risk factor for the occurrence of a postoperative complication (p < 0.01). None of the comorbidities was associated with the occurrence of a severe complication (CD ≥ 3b). In the supplementary material, three tables (S2-S4 Tables) show the distribution of postoperative complications by urinary diversion and surgical approach. Logistic regression was performed for variables with both outcome categories, but no statistically significant associations were found (S5 and S6 Tables).

**Table 3:** The most common postoperative complications of both groups 30 days after RC.

Complication	Interventional group	Non-interventional group
Gastrointestinal, n^a^ (%)	12 (23.5)	12 (23.5)
Cardiopulmonary, n^a^ (%)	8 (15.7)	15 (29.4)
Infectious, n^a^ (%)	11 (21.6)	10 (19.6)
Wound/skin complications, n^a^ (%)	5 (9.8)	5 (9.8)
Transfusions, n^a^ (%)	24 (47.1)	21 (41.2)
Clavien Dindo ≥ 3b, n^a^ (%)	8 (15.7)	9 (17.6)

^a^Numbers reflect the number of patients (percentages).

**Table 4:** Multivariable Firth logistic regression of the comorbidities.

Comorbidity	Postoperative complications (any CD)		Postoperative complications CD ≥ 3b	
	OR (95% CI)	P-value	OR (95% CI)	P-value
Cardiac infarct	0.503 (0.002-97.071)	0.746	0.231 (0.002-2.758)	0.278
Heart failure	1.225 (0.138-18.555)	0.860	1.330 (0.166-9.150)	0.774
Coronary artery disease	23.811 (2.150-4668.831)	0.005*	1.963 (0.372-9.618)	0.411
Arrhythmia	0.862 (0.181-3.946)	0.842	0.899 (0.201-3.447)	0.880
Cardiomyopathy	0.305 (0.001-158.016)	0.677	1.254 (0.066-23.493)	0.873
Hypertension	1.916 (0.664-6.070)	0.232	1.346 (0.392-5.107)	0.639
Peripheral occlusive arterial disease	0.249 (0.023-1.522)	0.134	0.198 (0.002-1.615)	0.151
Cerebrovascular disease	3.291 (0.243-512.075)	0.404	1.591 (0.181-13.646)	0.664
Chronic pulmonary disease	0.663 (0.213-2.054)	0.472	1.244 (0.339-4.159)	0.730
Diabetes mellitus	0.519 (0.116-2.225)	0.371	2.182 (0.575-8.010)	0.243
Hemi-/paraplegia	4.723 (0.265-921.570)	0.333	1.042 (0.006-17.830)	0.981
Renal insufficiency	0.563 (0.053-7.514)	0.626	1.040 (0.121-6.219)	0.968
Other tumor disease	0.504 (0.186-1.384)	0.179	1.086 (0.354-3.232)	0.882
Alcohol abuse	0.608 (0.080-4.398)	0.607	3.683 (0.493-25.879)	0.192
Smoking	1.364 (0.489-3.898)	0.550	1.072 (0.327-3.751)	0.909

CD, Clavien Dindo; OR, odds ratio; CI, confidence interval; * p < 0.05.

### Final histopathology and oncologic outcome

**Fig 2:**
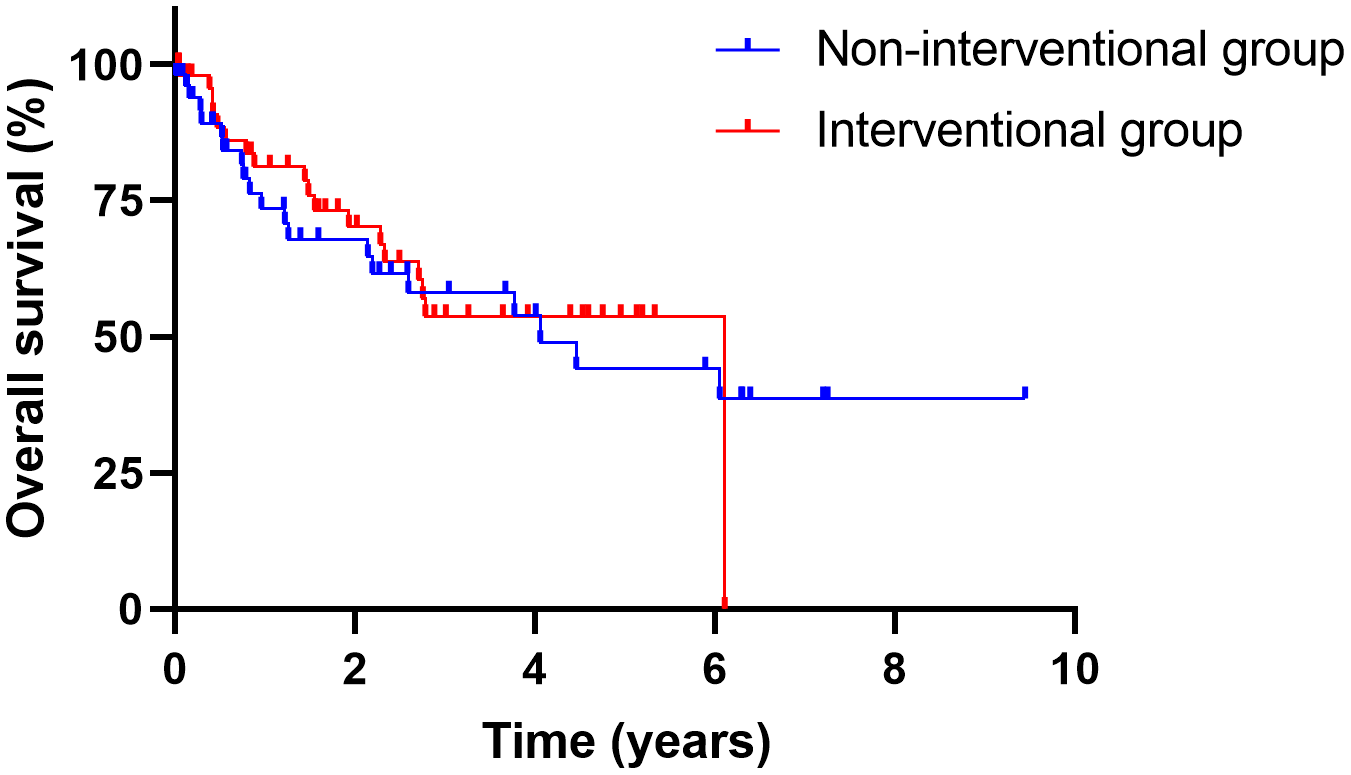
Kaplan-Meier curves for OS. Kaplan-Meier curves displaying overall survival after RC for the interventional group (red line) and for the non-interventional group (blue line).

**Fig 3:**
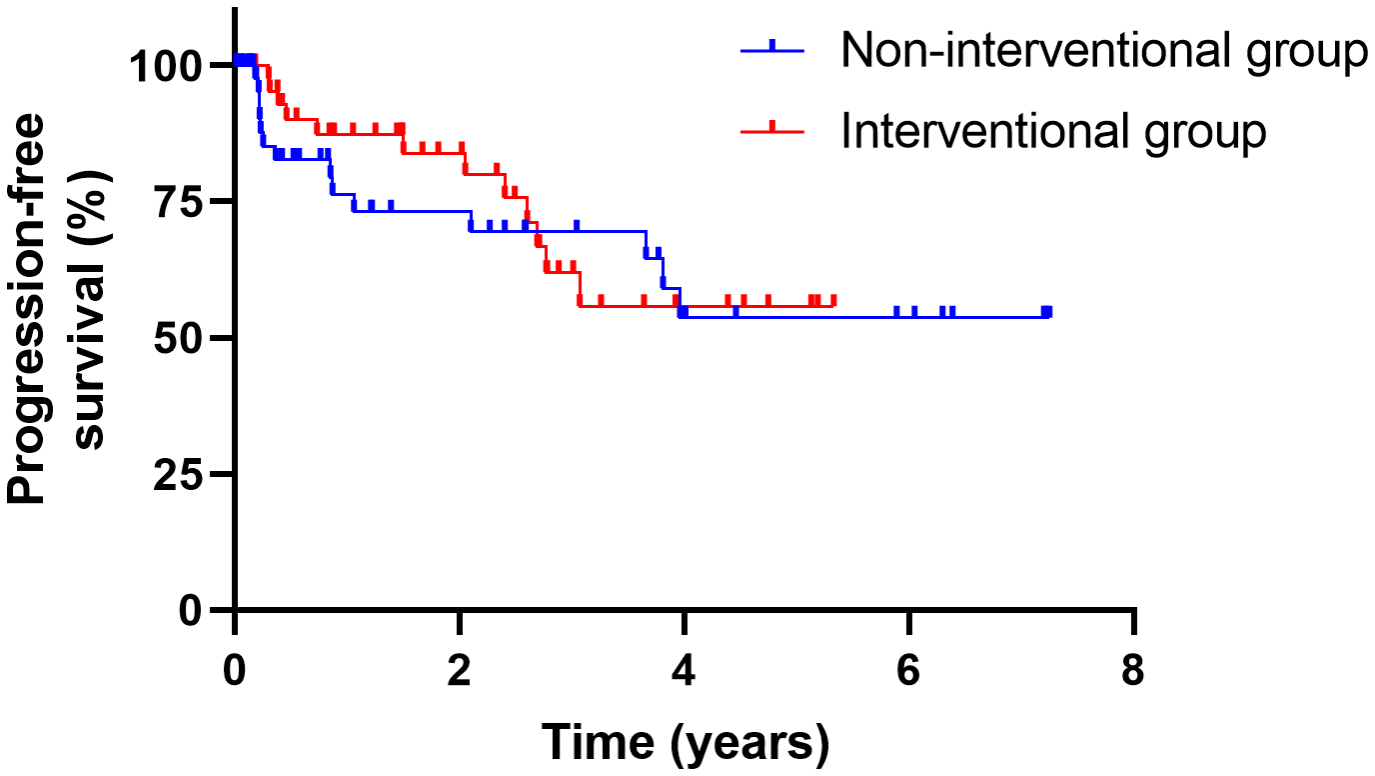
Kaplan-Meier curves for PFS. Kaplan-Meier curves showing progression-free survival after RC for the interventional (red line) and for the non-interventional group (blue line).

In the interventional group, the final histopathology results according to TNM classification after RC were: ypT0 in nine patients (17.6%), pTa/pTis in ten patients (19.6%), pT1 in four patients (7.8%), and ≥ pT2 in 27 patients (52.9%). There were eleven patients (21.6%) with pN+ disease. Twelve patients developed metastatic disease (23.5%) during follow-up, and two patients (3.9%) received palliative chemotherapy. In the non-interventional group, five patients (9.8%) had a pT0 stage, six patients (11.8%) had a pTis/pTa stage, seven patients (13.7%) had a pT1 stage, and a T-stage of ≥ pT2 was documented in 31 patients (60.8%) in the final histopathology report after RC. Nine patients were pN+ (17.6%), and 13 patients (25.5%) developed metastatic disease. In this group, four patients (7.8%) were treated with palliative chemotherapy. The TNM stage was analyzed as a covariate, with no significant difference observed between the two groups. The oncologic parameters are listed in Table 5. The mean follow-up period of patients in the interventional group was 24.9 months (SD: 20.8), with twelve relapses (23.5%) and 18 deaths (35.3%). In the non-interventional group, the mean follow-up period was 28.0 months (SD: 30.1), with 14 patients (27.5%) who experienced a relapse and 20 deaths (39.2%). There was no significant difference between the two groups in terms of OS (p = 0.76) or PFS (p = 0.65) (Figs 2 and 3).

**Table 5:** Oncologic parameters of both groups.

	Interventional group	Non-interventional group
Pathological T-stage at RC, n^a^ (%)		
pT0	9 (17.6)	5 (9.8)
pTa/pTis	10 (19.6)	6 (11.8)
pT1	4 (7.8)	7 (13.7)
pT2	9 (17.6)	5 (9.8)
pT3	12 (23.5)	15 (29.4)
pT4	6 (11.8)	11 (21.6)
n.a.	1 (2.0)	2 (3.9)
Pathological N-stage at RC, n^a^ (%)		
pN0	35 (68.6)	31 (60.8)
pNx	2 (3.9)	4 (7.8)
pN1	2 (3.9)	3 (5.9)
pN2	7 (13.7)	5 (9.8)
pN3	2 (3.9)	1 (2.0)
n.a.	3 (5.9)	7 (13.7)
Metastatic disease during FU (%)	12 (23.5)	13 (25.5)
Chemotherapy, n^a^ (%)		
neoadjuvant	4 (7.8)	3 (5.9)
adjuvant	4 (7.8)	6 (11.8)
palliative	2 (3.9)	4 (7.8)
Relapses, n^a^ (%)	12 (23.5)	14 (27.5)
Overall-mortality, n^a^ (%)	18 (35.3)	20 (39.2)
Follow-up period, months (SD)	24.9 (20.8)	28.0 (30.1)

^a^Numbers reflect the number of patients (percentages); RC, radical cystectomy; SD, standard deviation; FU, follow-up

Regarding the entire cohort, as expected, OS was significantly worse for tumors > pT2 compared with < pT2 (p < 0.05) and for N1 compared with N0/x (p = 0.02) in the univariate analysis. The association with >pT2 (p < 0.05) remained significant in the multivariable Cox regression model as well (S7 Table).

## Discussion

In patients with high-risk or very high-risk NMIBC, RC with curative intent remains a reasonable treatment option. International guidelines recommend RC after failure of intravesical BCG treatment or relapse during intravesical treatment. However, the indication for RC must be carefully evaluated, as it is a life-changing procedure associated with complications, including gastrointestinal complications in approximately 20% of cases, infectious complications in 17%, and ileus in 14% [5]. Our study focused on the impact of intravesical treatment in NMIBC patients receiving RC and its potential association with complications. Using a matched pair approach with clinically relevant features such as ASA-score, BMI, and age, we did not detect differences in postoperative complications. According to CD, there were also no statistically significant differences between the groups with complications ≥ 3b. Interestingly, these findings were independent of the final histopathology report, indicating that the local tumor burden did not significantly affect the feasibility of bladder removal. The most common complications in our study were gastrointestinal (23.5% in both groups), cardiopulmonary (15.7% vs. 29.4%), and infections (21.6% vs. 19.6%), which is comparable to data from a large meta-analysis by Katsimperis et al., including 44 studies in patients receiving RC for any histopathologic grade [5]. This aligns with other reports in which gastrointestinal complications (26.9%) were among the most common [11]. Due to comparable postoperative complications, intravesical treatment does not deteriorate the surrounding tissue of the bladder, which would affect the surgical procedure.

In another study by Haas et al., obesity, higher ASA-score, and medical drug anticoagulation were identified as risk factors for a higher rate of complications after RC for both NMIBC and MIBC patients [12]. On the contrary, a study by Adamczyk et al. analyzed 553 patients receiving RC for complications and comorbidities, and they concluded that the patients’ ASA scores did not increase the risk of complications after RC [13]. In concordance with our results, their study did not reveal differences regarding the final TNM stage. We identified CAD as a risk factor significantly associated with the incidence of postoperative complications (p = 0.005). In another recently published study, Kayra et al. also found that CAD was associated with a significantly higher rate of postoperative complications. However, in contrast to our results, this was also the case for higher BMI, ASA-score, hypertension, and diabetes mellitus [14].

Our data provide two main conclusions: First, intravesical treatment does not negatively influence the postoperative clinical course, as measured by postoperative complications, which was the study’s main hypothesis. Therefore, local inflammatory reactions and deterioration of the surrounding bladder tissue after repetitive transurethral resection, intravesical treatment, as well as tumor stage at final histopathology did not increase the rate of postoperative complications. Typically, the side effects of intravesical treatment occur at the beginning of the treatment and do not increase with the number of applied cycles [15,16]. Still, the absence of symptoms is not necessarily a direct indicator of surgical operability or postoperative complications following multiple intravesical instillations, TUR-Bs, and subsequent RC.

Secondly, in our study, PFS and OS were similar in both groups, consistent with the retrospective study by Contieri et al. investigating NMIBC patients treated with BCG who underwent delayed versus immediate RC [6]. They reported a five-year cancer-specific mortality of 5.3% in BCG-pretreated patients versus 4.9% in those undergoing immediate RC without prior BCG, with no significant difference.

Notably, patients with a final histopathology of pT0 have better OS than patients with a priori > pT2 stage; therefore, one might expect to see differences in OS. In the interventional group, 18% had a pT0 stage in the final histopathology report, but only 10% in the non-interventional group. Regarding the ≥ pT2 stage, the percentages in the non-interventional group were higher (61%) than in the interventional group (53%). However, as mentioned before, we used the TNM stage as a covariate, which did not differ between the groups, resulting in non-significant differences in OS and PFS.

The main limitations of this study are related to the retrospective study design. First, the matching was performed manually, focusing on four matching criteria (ASA-score, BMI, age, and sex) that are relevant to the postoperative clinical course after RC [17–19]. TNM stage is another important clinical factor; however, since the results of TUR-B often do not accurately reflect the final TNM stage, we included this variable as a covariate rather than a matching criterion, as it is not reliably available preoperatively. The manual matching method was deliberately chosen to ensure that each criterion was matched with a corresponding counterpart based on the predefined parameters. While propensity score matching may be more suitable for larger patient cohorts to ensure comparable group sizes and include additional variables, we considered fifty-one matched pairs per group to be both feasible and representative, given the high surgical volume at our center. Concerning urinary diversion, the distribution of ileal conduits and ureterocutaneostomies is approximately balanced. However, it is important to note that the number of neobladders, representing one of the most complex types of urinary diversion, is overrepresented in the non-interventional group, potentially leading to more complications in that group (ten vs. three in the interventional group). Still, the overall incidence of postoperative gastrointestinal complications was similar in both groups, suggesting no association with the operative procedure.

Secondly, intravesical treatment was also retrospectively assessed and is typically given by the outpatient practitioner. Therefore, we cannot evaluate if all intravesical regimes were given properly. Third, the postoperative standard changed during the screened period due to the implementation of the ERAS protocol, which might have impacted postoperative outcomes [20]. There is evidence that patients undergoing RC with an ERAS protocol suffer less from wound healing disorders, fever, and thrombosis [21]. This improvement in perioperative care is expected to result in fewer complications for patients treated according to this protocol.

In conclusion, complications in patients with NMIBC, whether they received prior intravesical treatment or not before RC, were similarly distributed, along with comparable oncologic outcome parameters. Therefore, intravesical treatment in high- or very high-risk NMIBC populations was not associated with differences in oncologic or perioperative complications. Our data may be helpful in the discussion between clinicians and patients regarding the safety of intravesical treatment, optimal timing of radical cystectomy, and potential postoperative complications, in consideration of tumor stage and the patient’s overall condition.

## Supporting information

S1 Table Patient characteristics of the full urothelial carcinoma cohort. (PDF)

S2 Table Postoperative complications by type of urinary diversion (incontinent vs continent). Distribution of postoperative complications stratified by type of urinary diversion (incontinent and continent diversion).
(PDF)

S3 Table Postoperative complications by type of urinary diversion. Distribution of postoperative complications stratified by type of urinary diversion (ureterocutaneostomy, conduit, and continent diversion).
(PDF)

S4 Table Postoperative complications by surgical approach. Distribution of postoperative complications stratified by type of surgical approach (robotic-assisted and open).
(PDF)

S5 Table Logistic regression analysis of postoperative complications by type of urinary diversion. Results of logistic regression analysis evaluating the association between type of urinary diversion (continent vs. incontinent) and postoperative complications.
(PDF)

S6 Table Logistic regression analysis of postoperative complications by surgical approach. Results of logistic regression analysis evaluating the association between surgical approach (robotic-assisted and open) and postoperative complications.
(PDF)

S7 Table Cox regression for T- and N-stage at RC. Cox proportional hazards regression analysis for overall survival according to T- and N-stage.
(PDF)

S1 Dataset Minimal dataset.
(XLSX)
