# Supplementary material for: Complications and oncologic outcome in bladder cancer patients receiving radical cystectomy after intravesical instillation treatment
Source: PLoS One. 2025 Dec 5;20(12):e0337644. doi: 10.1371/journal.pone.0337644 (PMC12680265; doi:10.1371/journal.pone.0337644)
Supplement: S1 Table — (PDF) [file pone.0337644.s001.pdf]

S1 Table. Patient characteristics of the full urothelial carcinoma cohort

	Full cohort (n[a] = 366)
Mean age, years (SD)	70 (9.3)
Male, n[a] (%)	287 (78.4)
Women, n[a] (%)	79 (21.6)
Mean BMI (SD)	26.8 (4.7)
BMI Cluster, n[a] (%)	
1	7 (1.9)
2	129 (35.2)
3	149 (40.7)
4	65 (17.8)
5	13 (3.6)
6	3 (0.8)
ASA-score, n[a] (%)	
1	1 (0.3)
2	145 (39.6)
3	188 (51.4)
4	23 (6.3)
n.a.	9 (2.5)

[a]Numbers reflect the number of patients (percentages); SD, standard deviation; BMI, body mass index; ASA, American Society of Anesthesiologists; n.a., not available
