# Supplementary material for: Complications and oncologic outcome in bladder cancer patients receiving radical cystectomy after intravesical instillation treatment
Source: PLoS One. 2025 Dec 5;20(12):e0337644. doi: 10.1371/journal.pone.0337644 (PMC12680265; doi:10.1371/journal.pone.0337644)
Supplement: S2 Table — Distribution of postoperative complications stratified by type of urinary diversion (incontinent and continent diversion). (PDF) [file pone.0337644.s002.pdf]

S2 Table. Postoperative complications by type of urinary diversion (incontinent vs continent)

Complication	Urinary diversion	
	Incontinent	Continent
No gastrointestinal, n[a]	64	13
Gastrointestinal, n[a]	20	4
No cardiopulmonary, n[a]	64	14
Cardiopulmonary, n[a]	20	3
No infectious, n[a]	69	11
Infectious, n[a]	15	6
No wound/skin complications, n[a]	74	17
Wound/skin complications, n[a]	10	0
No transfusions, n[a]	44	13
Transfusions, n[a]	40	4
No Clavien Dindo ≥3b, n[a]	68	16
Clavien Dindo ≥3b, n[a]	16	1

[a]Numbers reflect the number of patients
