# Supplementary material for: Complications and oncologic outcome in bladder cancer patients receiving radical cystectomy after intravesical instillation treatment
Source: PLoS One. 2025 Dec 5;20(12):e0337644. doi: 10.1371/journal.pone.0337644 (PMC12680265; doi:10.1371/journal.pone.0337644)
Supplement: S3 Table — Distribution of postoperative complications stratified by type of urinary diversion (ureterocutaneostomy, conduit, and continent diversion). (PDF) [file pone.0337644.s003.pdf]

S3 Table. Postoperative complications by type of urinary diversion

Complication	Urinary diversion		
	UCN	Conduit	Continent
No gastrointestinal, n[a]	5	59	13
Gastrointestinal, n[a]	0	20	4
No cardiopulmonary, n[a]	3	61	14
Cardiopulmonary, n[a]	2	18	3
No infectious, n[a]	4	65	11
Infectious, n[a]	1	14	6
No wound/skin complications, n[a]	5	69	17
Wound/skin complications, n[a]	0	10	0
No transfusions, n[a]	2	42	13
Transfusions, n[a]	3	37	4
No Clavien Dindo ≥3b, n[a]	5	63	16
Clavien Dindo ≥3b, n[a]	0	16	1

[a]Numbers reflect the number of patients; UCN, ureterocutaneostomy
