# Supplementary material for: Complications and oncologic outcome in bladder cancer patients receiving radical cystectomy after intravesical instillation treatment
Source: PLoS One. 2025 Dec 5;20(12):e0337644. doi: 10.1371/journal.pone.0337644 (PMC12680265; doi:10.1371/journal.pone.0337644)
Supplement: S4 Table — Distribution of postoperative complications stratified by type of surgical approach (robotic-assisted and open). (PDF) [file pone.0337644.s004.pdf]

S4 Table. Postoperative complications by surgical approach

Complication	Surgical approach	
	Robotic-assisted	Open
No gastrointestinal, n[a]	9	69
Gastrointestinal, n[a]	1	23
No cardiopulmonary, n[a]	10	69
Cardiopulmonary, n[a]	0	23
No infectious, n[a]	8	73
Infectious, n[a]	2	19
No wound/skin complications, n[a]	10	82
Wound/skin complications, n[a]	0	10
No transfusions, n[a]	10	47
Transfusions, n[a]	0	45
No Clavien Dindo ≥3b, n[a]	10	75
Clavien Dindo ≥3b, n[a]	0	17

[a]Numbers reflect the number of patients
