# Supplementary material for: Complications and oncologic outcome in bladder cancer patients receiving radical cystectomy after intravesical instillation treatment
Source: PLoS One. 2025 Dec 5;20(12):e0337644. doi: 10.1371/journal.pone.0337644 (PMC12680265; doi:10.1371/journal.pone.0337644)
Supplement: S5 Table — Results of logistic regression analysis evaluating the association between type of urinary diversion (continent vs. incontinent) and postoperative complications. (PDF) [file pone.0337644.s005.pdf]

S5 Table. Logistic regression analysis of postoperative complications by type of urinary diversion

Complication	Urinary diversion (continent vs incontinent)	
	OR (95% CI)	P-value
Gastrointestinal	0.985 (0.288-3-362)	0.980
Cardiopulmonary	0.686 (0.179-2.630)	0.582
Infectious	2.509 (0.802-7.851)	0.114
Wound/skin complications	-	-
Transfusions	0.338 (0.102-1.123)	0.077
Clavien Dindo ≥3b	0.266 (0.033-2.153)	0.214

OR, odds ratio; CI, confidence interval
