# Supplementary material for: Complications and oncologic outcome in bladder cancer patients receiving radical cystectomy after intravesical instillation treatment
Source: PLoS One. 2025 Dec 5;20(12):e0337644. doi: 10.1371/journal.pone.0337644 (PMC12680265; doi:10.1371/journal.pone.0337644)
Supplement: S6 Table — Results of logistic regression analysis evaluating the association between surgical approach (robotic-assisted and open) and postoperative complications. (PDF) [file pone.0337644.s006.pdf]

S6 Table. Logistic regression analysis of postoperative complications by surgical approach

Complication	Surgical approach (robotic-assisted vs open)	
	OR (95% CI)	P-value
Gastrointestinal	3.000 (0.360-24.973)	0.310
Cardiopulmonary	-	-
Infectious	1.041 (0.204-5.312)	0.961
Wound/skin complications	-	-
Transfusions	-	-
Clavien Dindo ≥3b	-	-

OR, odds ratio; CI, confidence interval
