# Supplementary material for: Complications and oncologic outcome in bladder cancer patients receiving radical cystectomy after intravesical instillation treatment
Source: PLoS One. 2025 Dec 5;20(12):e0337644. doi: 10.1371/journal.pone.0337644 (PMC12680265; doi:10.1371/journal.pone.0337644)
Supplement: S7 Table — Cox proportional hazards regression analysis for overall survival according to T- and N-stage. (PDF) [file pone.0337644.s007.pdf]

S7 Table. Cox regression for T- and N-stage at RC

T/N-stage at RC	HR (95% CI), univariate	P-value	HR (95% CI), multivariable	P-value
T2 vs. <T2	2.069 (0.596-7.177)	0.252	2.115 (0.607-7.368)	0.240
>T2 vs. <T2	4.485 (1.724-11.668)	**0.002***	3.878 (1.480-10.164)	**0.006***
N1 vs. N0/x	2.320 (1.148-4.690)	**0.019***	1.907 (0.921-3.952)	0.082

RC, radical cystectomy; HR, hazard ratio; CI, confidence interval; * $p < 0.05$
